# Supplementary material for: Splice-Junction-Based Mapping of Alternative Isoforms in the Human Proteome
Source: Cell Rep. Author manuscript; Available in PMC 2020 Jan 15. (PMC6961840; doi:10.1016/j.celrep.2019.11.026)
Supplement: 2 [file NIHMS1546469-supplement-2.zip › DF1/PXD006675/RightAtrium/RightAtrium_39_TTN_ENMATLMVLEPPK.pdf]

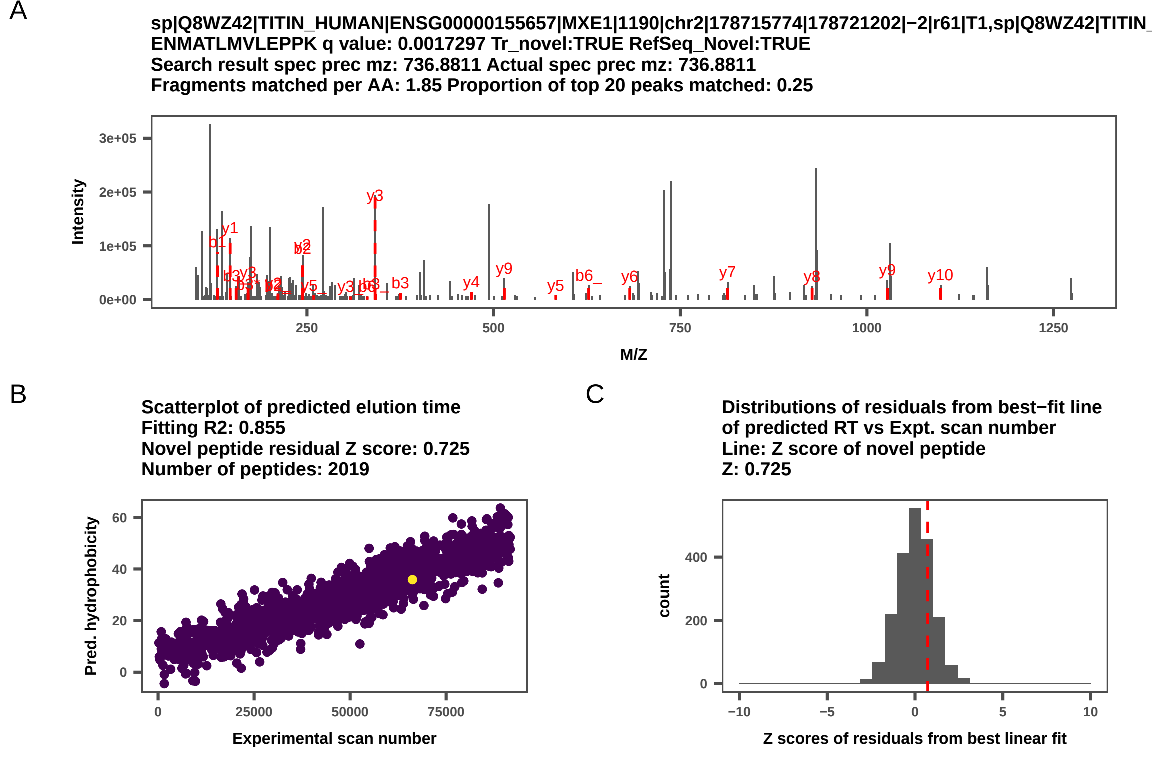
A
sp|Q8WZ42|TITIN_HUMAN|ENSG00000155657|MXE1|1190|chr2|178715774|178721202|−2|r61|T1,sp|Q8WZ42|TITIN_
ENMATLMVLEPPK q value: 0.0017297 Tr_novel:TRUE RefSeq_Novel:TRUE
Search result spec prec mz: 736.8811 Actual spec prec mz: 736.8811
Fragments matched per AA: 1.85 Proportion of top 20 peaks matched: 0.25
Intensity
3e+05
2e+05
1e+05
0e+00
250
500
750
1000
1250
M/Z
y1
b1
y3
y9
y4
y5
b6_
y6
y7
y8
y9
y10
b3
B
Scatterplot of predicted elution time
Fitting R2: 0.855
Novel peptide residual Z score: 0.725
Number of peptides: 2019
Pred. hydrophobicity
60
40
20
0
0
25000
50000
75000
Experimental scan number
C
Distributions of residuals from best−fit line
of predicted RT vs Expt. scan number
Line: Z score of novel peptide
Z: 0.725
count
400
200
0
−10
−5
0
5
10
Z scores of residuals from best linear fit
